# Le cancer en Mauritanie : résultats sur 10 ans du registre hospitalier de Nouakchott

**DOI:** 10.11604/pamj.2013.14.149.2565

**Published:** 2013-04-14

**Authors:** Nacer Dine Ould Mohamed Baba, Catherine Sauvaget

**Affiliations:** 1 Service Anatomie et Cytologie Pathologiques, Centre Hospitalier National, Nouakchott, Mauritanie; 2 Groupe de dépistage, Section de prévention et détection précoce, Centre International de Recherche contre le Cancer, Lyon, France

**Keywords:** Cancer, anatomie pathologie, épidémiologie descriptive, Mauritanie, Cancer, Anatomy pathology, descriptive epidemiology, Mauritania

## Abstract

Le fardeau du cancer reste mal connu en Mauritanie. Il n'est basé que sur des extrapolations de l'incidence des cancers des pays avoisinants. Les données du registre de l'hôpital national permettent de décrire les cas avec un diagnostic histologique. Tous les cas de cancers enregistrés par le service d'anatomo-pathologie de l'hôpital national de Nouakchott de 2000 à 2009 ont été analysés. En 10 ans, 3305 prélèvements histologiques ont été analysés (hommes:42%, femmes:58%). Chez l'homme, le cancer le plus fréquemment analysé était le cancer de la peau (218 cas au total, 189 cas en excluant mélanome), suivi de la prostate (203), des cancers digestifs (179, colorectal et ‘sophage), et des lymphomes (151). Chez la femme, un quart des biopsies était des cancers du sein (485), suivi du col utérin (344), de la sphère gynécologique (218, ovaire et corps utérin), et de la peau (114). Les cancers du foie, du poumon ou de la vessie étaient peu fréquents. Ces résultats ne reflètent pas l'incidence ni l'actuel fardeau du cancer en Mauritanie puisque de nombreux patients diagnostiqués avec un cancer ne reçoivent pas d'examen anatomopathologique. Si, comme dans les pays avoisinants du Maroc et du Mali, les cancers du col et du sein sont les pathologies les plus fréquentes chez la femme, la distribution des cancers chez l'homme dans ce registre hospitalier diffère des résultats des registres de population du Maroc et du Mali où les cancers du poumon, du foie, de la prostate et de la vessie dominent.

## Introduction

Selon un rapport récent de l'Organisation Mondiale de la Santé (OMS) [1], le cancer représente 5% des causes de décès en Mauritanie. La majorité des décès (59%) sont dus aux maladies transmissibles et maternelles et infantiles. Jusqu’à récemment, le cancer ne figurait pas sur l'agenda national; la Mauritanie ne possédant pas de plan cancer, ni de programme de détection précoce, ni de registre national des cas de cancer basé sur la population. Avec une population de 3,5 millions d'habitants, et une espérance de vie à la naissance de 57 ans pour les hommes et 60 ans pour les femmes, il a été estimé que 2000 nouveaux cas de cancers sont diagnostiqués chaque année dans le pays et 1500 personnes décèdent chaque année du cancer [2, 3]. Ces estimations sont basées sur l'incidence et la durée de survie par cancer des pays avoisinants.

Le pays possède un service d'anatomie pathologique et de cytologie créé il y a une dizaine d'années au sein du centre hospitalier national de Nouakchott. Avec le récent développement de l'anatomie pathologique et la création de nouvelles structures hospitalières, des informations plus précises sur le nombre de cas et leur répartition s'avèrent dorénavant possibles. L'objectif de cette étude est de décrire les cas de cancers analysés par notre service entre 2000 et 2009, en termes de distribution et, pour les cancers les plus fréquents, de type histologique.

## Méthodes

Cette étude est basée sur l'ensemble des données du registre des cancers du centre hospitalier national de Nouakchott qui se trouve au sein du service d'anatomie pathologique et de cytologie du même établissement avec quelques données recueillies auprès des structures sanitaires privées et dans les hôpitaux étrangers qui accueillent des patients mauritaniens évacués pour y être traités. Nous avons analysé les cas enregistrés sur la période du 1er Janvier 2000 au 31 Décembre 2009. Ces données ont été croisées et complétées avec celles des cliniciens. Il s'agissait de cancers primaires uniquement. Pour chaque cas, le diagnostic est retenu à l'histologie, la classification utilisée est celle de l'OMS (classification internationale des maladies pour l'oncologie). Après élimination des doublons et vérification de la conformité du diagnostic avec la localisation, les cas sont entrés dans un fichier Excel. La distribution des localisations, ainsi que l'histologie des cinq principaux cancers sont rapportés chez les hommes et les femmes séparément.

## Résultats

Un total de 3534 prélèvements histologiques furent reçus par le service pendant les 10 années d’étude; 3305 biopsies ayant été analysées et 224 étant inadéquates à cause d'information manquante sur la localisation ou le diagnostic. L'annexe 1 montre la distribution des cancers chez les hommes (1403 cas, 42%) et chez les femmes (1902 cas, 58%) en fonction du site du cancer, de l’âge moyen au diagnostic et de la tranche d’âge. Chez l'homme, le cancer le plus fréquemment biopsié était celui de la peau (2e position si exclusion des mélanomes), suivi de la prostate, des cancers digestifs, et des lymphomes. Les biopsies des poumons étaient en 8e position et de la sphère ORL en 10e. L’âge moyen à la biopsie était la quarantaine et la cinquantaine, sauf pour les cancers de la prostate à 71 ans. Seulement 3 cancers du foie ont été analysés. Chez la femme, un quart des biopsies était des cancers du sein, suivi du cancer du col utérin, de la sphère gynécologique (ovaire et corps utérin), de la peau, de la thyroïde et du système digestif. L’âge moyen à la biopsie était la trentaine et la quarantaine. L'annexe 2 montre la distribution histologique des cinq principaux cancers biopsiés chez l'homme et la femme. Chez l'homme, les cancers de la peau étaient essentiellement des carcinomes épidermoïdes et basocellulaires, les cancers de la prostate des adénocarcinomes, les lymphomes des lymphomes malins non hodgkiniens, les cancers colorectaux des adénocarcinomes, et les cancers de l'œsophage des carcinomes épidermoïdes. Chez la femme, les cancers du sein étaient principalement des carcinomes canalaires infiltrants, les cancers du col utérin des carcinomes épidermoïdes, ceux de la peau des carcinomes basocellulaires et épidermoïdes, un tiers des cancers ovariens étaient des adénocarcinomes et enfin les cancers de l'endomètre étaient surtout des adénocarcinomes.

## Discussion

Cette étude résume le travail d'une dizaine d'années du service d'anatomie pathologique, en décrivant les cancers avec un examen histologique. Les résultats ne reflètent pas l'incidence ni l'actuel fardeau du cancer en Mauritanie puisque de nombreux patients n'ont jamais été biopsiés.

Les cancers les plus fréquemment analysés étaient les cancers gynécologiques (sein et col utérin), la peau, les lymphomes, la prostate et les cancers digestifs. Par contre, les cancers dus au tabagisme (poumons, ORL, vessie) ou les cancers du foie étaient peu biopsiés.

La population mauritanienne est composée d'ethnies arabo-berbères et sub-sahariennes, avec des différences dans leurs modes de vie, leurs expositions aux facteurs de risque, leurs prédispositions génétiques. Lorsque l'on compare l'incidence publiée par les registres du cancer du voisinage avec les différentes ethnies, au Maroc (Grand Casablanca et Rabat) et au Mali [4–6], on observe des contrastes dans la fréquence des cancers chez l'homme et des similitudes chez la femme. Dans les 2 registres marocains, les cancers les plus fréquents chez l'homme sont le poumon et la prostate, suivis par le colon, la vessie et les lymphomes non-hodgkiens à Casablanca, ou suivis par la vessie, les lymphomes non-hodgkiens et l'estomac à Rabat. Par contre au Mali, le cancer le plus commun chez les hommes est le cancer du foie, suivi de l'estomac, la vessie, la prostate et le colon. Ces différentes localisations indiquent des différences dans l'exposition aux facteurs de risque: tabagisme, et vieillissement de la population au Maroc; essentiellement maladies virales, fungiques et parasitaires (virus hépatite B et C, aflatoxine, bilharziose), tabagisme et ‘ à un moindre niveau - vieillissement de la population au Mali.

En revanche, des ressemblances avec le Maroc et le Mali sont retrouvées dans la fréquence des cancers féminins. Les cancers du sein et du col utérin sont les plus fréquents; le cancer le plus commun étant le sein au Maroc et le col au Mali. Au Maroc, les autres principales localisations sont l'ovaire et la thyroïde, alors qu'au Mali il s'agit de l'estomac, du foie et de la vessie, reflétant de nouveau l'importance d'une étiologie virale, fungique et parasitaire pour ces 2 derniers sites.

Par conséquent, les résultats rapportés ici sous-estiment certainement le fardeau des cancers du poumon, foie, et vessie dans la population. La difficulté des prélèvements endobronchiques, hépatiques et vésicaux ont probablement contribué au faible taux des cancers retrouvés dans ces localisations.

## Conclusion

Une politique anti cancer est nécessaire en Mauritanie; celle-ci incluant la mise en place d'un registre national des cas de cancer basé sur la population afin de chiffrer et de suivre l’évolution du fardeau de cette maladie, l'implémentation de programmes de prévention primaire (lutte contre le tabagisme, vaccination contre le virus de l'hépatite B, vaccination contre le virus du papillome humain, éradication de la bilharziose), implémentation de programmes de lutte contre le cancer du col utérin et du sein par l'intermédiaire d'un dépistage précoce. Quant à la prévention tertiaire, elle commence à se mettre en place avec la création récente de nouvelles structures de prise en charge des malades cancéreux à Nouakchott.

## Annexes

**Annexe 1:** Description des cas par site, chez l'homme et la femme

**Annexe 2:** Distribution histologique des cinq principaux cancers chez l'homme et la femme
